# P02.141. Effects of a low omega-6 and high omega-3 diet on inflammatory gene expression in patients with chronic daily headaches

**DOI:** 10.1186/1472-6882-12-S1-P197

**Published:** 2012-06-12

**Authors:** S Smith, C Ramsden, S Gaylord, K Faurot, C Lynch, J Mann

**Affiliations:** 1 University of North Carolina at Chapel Hill Medical School, Chapel Hill, USA; 2 NIH Section on Nutritional Neurosciences, Bethesda, USA

## Purpose

Approximately 10 million U.S. adults suffer from chronic daily headache (CDH), resulting in major disability and impaired quality of life. Recent evidence suggests that the cause of pain may be related to inflammation. Dietary interventions that reduce the inflammatory response could help eliminate the development of pain in chronic pain syndromes such as CDH. The goal of this pilot study was to assess whether dietary modifications, designed to decrease dietary omega-6 fatty acids and increase omega-3 fatty acids, influence the expression of genes that are involved in inflammation in patients with CDH.

## Methods

Patients with CDH were placed on a low omega-6 diet or a low omega-6 plus high omega-3 diet for 12-weeks. Peripheral blood mononuclear cells were isolated from whole blood before and after the 12-week intervention. Real-time quantitative PCR was utilized to measure changes in gene expression. The expression of inflammatory cytokines and chemokines, inflammatory enzymes and genes involved in the NF-κB signaling pathway, were analyzed.

## Results

Both the low omega-6 and the low omega-6 plus high omega-3 diets, after 12-weeks, significantly decreased the expression of pro-inflammatory cytokines and chemokines. Additionally, both diets decreased the expression of pro-inflammatory enzymes and genes that are involved in the NF-κB signaling pathway.

## Conclusion

These data reveal that decreasing omega-6 fatty acids may exert clinical effects via the capacity to regulate the expression of pro-inflammatory mediators. Since the study is still on-going, and hence still blinded, we do not know which diet the patients were receiving, just that both diets decrease pro-inflammatory gene expression. Following completion of the study, we will be able to compare the effects of the low omega-6 vs. low omega-6 plus high omega-3 diets on inflammatory gene expression to determine which diet, if either, was more effective in reducing expression.

